# Lung Function Course of Patients With Pulmonary Fibrosis After Initiation of Anti‐Fibrotic Treatment: Real‐World Data From the Dutch National Registry

**DOI:** 10.1111/resp.70030

**Published:** 2025-03-23

**Authors:** Mark G. J. P. Platenburg, Gizal Nakshbandi, Catharina C. Moor, Aernoud A. van Batenburg, Rémy L. M. Mostard, Mareye Voortman, Linda A.A. Moonen, Nicolle Hekelaar, Maria J. Overbeek, Brigitte A.H.A. Bogaarts, Henk Kramer, Emiel. R. Marges, Bart B. Boerrigter, Paul Bresser, Eveline L. Schakenraad, Jan van der Maten, Niels C.A. van der Sloot, Stefan Walen, Pedro Miranda Afonso, Marlies S. Wijsenbeek, Jan C. Grutters

**Affiliations:** ^1^ ILD Centre of Excellence, Member of European Reference Network‐Lung St Antonius Hospital Nieuwegein the Netherlands; ^2^ Centre of Excellence for ILD and Sarcoidosis, Department of Respiratory Medicine Erasmus Medical Centre Rotterdam the Netherlands; ^3^ Department of Respiratory Medicine Zuyderland Medical Centre Heerlen the Netherlands; ^4^ Department of Respiratory Medicine Maastricht University Medical Centre (MUMC+) Maastricht the Netherlands; ^5^ Department of Pulmonology, Division of Heart & Lungs University Medical Centre Utrecht Utrecht the Netherlands; ^6^ Department of Pulmonary Medicine Rijnstate Hospital Arnhem the Netherlands; ^7^ Department of Pulmonary Medicine Medisch Spectrum Twente Enschede the Netherlands; ^8^ Department of Pulmonary Medicine Haaglanden Medical Centre The Hague the Netherlands; ^9^ Department of Pulmonary Diseases VieCuri Medical Centre Venlo the Netherlands; ^10^ Department of Pulmonary Medicine Martini Hospital Groningen the Netherlands; ^11^ Department of Respiratory Medicine Leiden University Medical Centre Leiden the Netherlands; ^12^ Department of Pulmonary Medicine, Centre of Excellence for Interstitial Lung Diseases and Sarcoidosis Amsterdam University Medical Centre, VUMC Amsterdam the Netherlands; ^13^ ILD Centre of Excellence, Department of Respiratory Medicine OLVG Amsterdam the Netherlands; ^14^ Department of Pulmonary Medicine Catharina Hospital Eindhoven the Netherlands; ^15^ Department of Pulmonary Medicine Medical Centre Leeuwarden Leeuwarden the Netherlands; ^16^ Department of Pulmonary Medicine Jeroen Bosch Hospital Hertogenbosch the Netherlands; ^17^ Department of Pulmonology Isala Zwolle the Netherlands; ^18^ Department of Biostatistics Erasmus Medical Centre Rotterdam the Netherlands; ^19^ Department of Epidemiology Erasmus Medical Centre Rotterdam the Netherlands

**Keywords:** anti‐fibrotic medication, idiopathic pulmonary fibrosis, lung function, progressive pulmonary fibrosis

## Abstract

**Background and Objective:**

Real‐world data on lung function course of patients with progressive pulmonary fibrosis (PPF) treated with anti‐fibrotic medication are limited. We evaluated forced vital capacity (FVC) decline in patients with PPF and idiopathic pulmonary fibrosis (IPF) who started anti‐fibrotic treatment.

**Methods:**

This was a nationwide multi‐centre registry study in 16 hospitals throughout the Netherlands. Patients treated with anti‐fibrotic medication, with at least two in‐hospital pulmonary function tests before and after the initiation of anti‐fibrotic treatment, were included. Linear mixed‐effects modelling was used to analyse lung function trajectories 1 year before and after the start of anti‐fibrotic treatment.

**Results:**

Data from 538 patients (*n =* 142 with PPF, *n =* 396 with IPF) were analysed. In PPF, the mean annualised FVC decline was 412 mL (95% confidence interval [CI]: 308–517 mL) before the initiation of anti‐fibrotic treatment, and 18 mL (95% CI: 9–124 mL) in the first year after. The corresponding declines for IPF were 158 mL (95% CI: 78–239 mL) and 38 mL (95% CI: 24–101 mL). In both groups, treatment significantly slowed down FVC decline, although the change was larger in the PPF group (*p* = 0.0006). In the first year after treatment initiation, 23.9% of patients with PPF and 28.0% with IPF had disease progression.

**Conclusion:**

The FVC decline significantly slowed after the initiation of treatment for both IPF and PPF. Nevertheless, a significant proportion of patients exhibited disease progression, despite the start of anti‐fibrotic treatment. Early identification of these patients is crucial for treatment adaptations and inclusion in clinical trials.

## Abstract 2

This nationwide registry study shows reduced lung function decline in progressive pulmonary fibrosis in the first year after anti‐fibrotic treatment initiation. However, almost a quarter of patients progresses within one year. These patients should be identified to offer participation in clinical trials with novel compounds on top of anti‐fibrotic treatment.

**Summary**

- This nationwide registry study shows reduced lung function decline in progressive pulmonary fibrosis in the first year after anti‐fibrotic treatment initiation.
- However, almost a quarter of patients progresses within one year.
- These patients should be identified to offer participation in clinical trials with novel compounds on top of anti‐fibrotic treatment.

## 1 Introduction

Pulmonary fibrosis is characterised by scarring of lung tissue causing irreversible lung function loss, with idiopathic pulmonary fibrosis (IPF) being the paradigmatic presentation of the disease with a progressive disease course and poor outcomes [1, 2]. Other forms of pulmonary fibrosis may display a similar progressive nature as witnessed in IPF, named progressive pulmonary fibrosis (PPF) in the recent ATS/ERS/JRS/ALAT guideline [3].

Clinical trials have demonstrated beneficial effects of anti‐fibrotic treatment in patients with PPF, comparable with IPF [4, 5, 6, 7, 8]. This has led to the approval of nintedanib for PPF and (conditional) recommendation in the international treatment guideline [3]. Anti‐fibrotic treatment is initiated when patients are progressive, despite standard care, which is often treatment with immunosuppressive medication [9, 10, 11]. In addition to the evidence from these clinical trials, there are some real‐world data on the beneficial effect of anti‐fibrotic drugs on the disease course in PPF from small studies [12, 13]. Although anti‐fibrotic treatment slows down disease progression, it does not completely stop or reverse the decline, and novel therapeutic approaches are still needed. The design and patient inclusion for clinical trials of novel compounds in IPF and PPF are challenging in the current treatment era. Data on the efficacy of anti‐fibrotic medication in daily clinical practice and the decline in lung function when using anti‐fibrotics are still scarce for PPF, whilst these insights could help guide study design.

A nationwide registry for patients with pulmonary fibrosis, known as The Dutch Society for Chest Physicians (NVALT) registry, has been established in the Netherlands. A unique feature of this registry is that all patients have been discussed in a multi‐disciplinary team (MDT) meeting with an expert centre for interstitial lung diseases. One of the primary objectives of this registry is to acquire real‐world data on treatment and disease behaviour in various forms of pulmonary fibrosis, aiming to enhance future disease management strategies and research.

In the current analysis, we evaluated the lung function trajectory in patients with PPF and IPF before and after the initiation of anti‐fibrotic treatment.

## 2 Methods

### 2.1 Registry Design

The NVALT registry study encompasses a nationwide cohort of patients with PPF and IPF from 16 hospitals throughout the Netherlands, including all but one of the hospitals treating patients with pulmonary fibrosis. All patients included in the registry were diagnosed with an interstitial lung disease in an MDT meeting at one of the four expert centres in the Netherlands according to current guidelines [3]. Patient data were stored in a secured cloud‐based clinical data management system (REDCap, version: 13.7.22, REDCap Consortium, Fort Lauderdale, United States) that is compliant with all safety regulations.

### 2.2 Study Cohort

For these analyses, we used the data of patients who were included in the registry from June 2022 until April 2023 and used anti‐fibrotic medication. Both incident and prevalent cases were included as patients with PPF started with anti‐fibrotics between 2017 (clinical trial setting) and March 2023. Patients underwent pulmonary function tests at an outpatient clinic every 3–6 months, including forced vital capacity (FVC) and diffusing capacity of the lung for carbon monoxide (DLCO) tests as part of regular care.

All available in‐hospital pulmonary function tests from 1 year before to 1 year after starting anti‐fibrotic treatment were used to model lung function courses over time. Initiation of anti‐fibrotic treatment was considered as the baseline for all patients. Only patients with at least two FVC measurements before and after starting treatment were included in further analyses.

### 2.3 Statistical Analysis

Descriptive statistics were used to analyse baseline characteristics. Differences in baseline characteristics between PPF and IPF were analysed using a Student's *t*‐test for normally distributed continuous data and a Wilcoxon rank‐sum test for non‐normally distributed continuous data. The chi‐square test was used to assess differences between categorical variables. Two linear mixed‐effect models were used to evaluate the change in percent predicted FVC, measured in both percent predicted and in litres, and DLCO in percent predicted 1 year before and after the start of treatment. The model included a linear effect of time since initiation of anti‐fibrotic treatment (days), use of anti‐fibrotic treatment (Yes/No), and patient group (PPF/IPF) as fixed effects. Time (days) and intercept were included as random effects. The model allowed for a difference in intercept and slope before and after starting anti‐fibrotic treatment. To compare the lung function trajectories in IPF and PPF, the model further allowed the changes to be different for both these patient groups. We performed a likelihood‐ratio test to compare the slopes before and after initiation of anti‐fibrotic treatment in both patient groups in the model. We used the physiological criteria for disease progression from the recent ATS/ERS/JRS/ALAT guidelines (absolute decline of ≥ 5% predicted in FVC and ≥ 10% predicted in DLCO within 1 year of follow up) to define the proportion of patients who experienced disease progression within the first year of anti‐fibrotic treatment [3]. All analyses were performed using R version 4.3.2. (R Foundation, Vienna, Austria).

## 3 Results

### 3.1 Patient Demographics

A total of 538 patients were included in this study (PPF: *n =* 142; IPF: *n =* 396). The median age was 71 years (interquartile range [IQR]: 66–76), and 78% were male. The most frequent diagnoses in the PPF group were connective tissue disease‐associated interstitial lung disease (CTD‐ILD: 32%) and fibrotic hypersensitivity pneumonitis (fHP: 18%). An overview of the baseline characteristics is shown in Table 1.

**TABLE 1:** Baseline characteristics.

	IPF (*n =* 396)	PPF (*n =* 142)	*p*
Median age at diagnosis (IQR)	70.0 (66.3–75.2)	64.9 (57.3–73.0)	< 0.001
Male sex (%)	326 (82.3)	96 (67.6)	< 0.001
Current or former smoker (%)	320 (80.8)	100 (70.4)	0.046
Diagnosis, *n* (%)			
		CTD‐ILD (%)	46 (32.4)
		FHP (%)	26 (18.3)
		iNSIP (%)	26 (18.3)
		Unclassifiable ILD (%)	15 (10.6)
		CPFE (%)	7 (4.9)
		Sarcoidosis (%)	6 (4.2)
		Other (%)	16 (11.2)
Immunosuppressive treatment, *n* (%)			
		Prednisone	29 (21.6)
		MMF	14 (9.9)
		Prednisone, MMF	13 (9.2)
		Azathioprine	7 (4.9)
		Methotrexate	5 (3.5)
		Prednisone, cyclophosphamide	5 (3.5)
		Other	15 (10.5)
		None	54 (38.0)

Abbreviations: CPFE, combined pulmonary fibrosis and emphysema; CTD‐ILD, connective tissue disease‐associated interstitial lung disease; DLCO, diffusing capacity of the lung for carbon monoxide; FHP, fibrotic hypersensitivity pneumonitis; FVC, forced vital capacity; INSIP, idiopathic non‐specific interstitial pneumonia; IPF, idiopathic pulmonary fibrosis; IQR, interquartile range; MMF, mycophenolate mofetil; PPF, progressive pulmonary fibrosis other than IPF.

At baseline, the majority of patients with PPF (88.7%) and IPF (59.3%) were treated with nintedanib. The remaining 11.3% of patients with PPF and 40.7% of patients with IPF were treated with pirfenidone. Patients with PPF initiated anti‐fibrotic treatment between January 2017 (in a clinical trial setting) and March 2023; patients with IPF started between March 2011 (in a clinical trial setting) and March 2023. The time from the initial MDT diagnosis of interstitial lung disease (ILD) to the initiation of anti‐fibrotic treatment differed significantly between patients with PPF and IPF, with a median difference of 1 year (*p <* 0.005). In patients with PPF, anti‐fibrotic treatment was started at a median of 401 days (IQR: 34–1379 days) after the MDT date (diagnosing ILD). In IPF, anti‐fibrotic medication was initiated at a median of 35 days following MDT (IQR: 18–109 days).

Almost two‐thirds (62.0%) of patients with PPF used concurrent immunosuppressive medication at baseline. At baseline, 40.1% of the PPF cohort were treated with one type of immunosuppressive medication, 21.2% with a combination of two types, and 0.7% with a combination of three. At 1 year, 18.5% (*n =* 23) of the patients with PPF had changed their immunosuppressive treatment, with 10 of the 23 patients discontinuing immunosuppressive medication.

### 3.2 Lung Function Trajectories Before and After Initiation of Anti‐Fibrotic Treatment

Patients with PPF had a mean FVC of 2.65 L (95% CI: 2.50–2.80) and 71.30% predicted (95% CI: 68.13%–74.47%) at baseline. Patients with IPF had a mean FVC of 3.13 L (95% CI: 3.04%–3.22%) and 83.3% predicted (95% CI: 81.3%–85.0%). The mean baseline DLCO was 40.8% predicted (95% CI: 38.4%–43.2%) in the PPF group, and 49.0% predicted (95% CI: 47.6%–50.5%) in the IPF group. The baseline FVC (*p <* 0.005) and DLCO (*p <* 0.005) were significantly lower in patients with PPF than in those with IPF (Table 2).

**TABLE 2:** Trajectory of forced vital capacity and diffusing capacity of the lung for carbon monoxide before and after the initiation of anti‐fibrotic treatment in patients with idiopathic pulmonary fibrosis and progressive pulmonary fibrosis.

	Pulmonary function at time of treatment initiation (mean, 95% CI)	Rate of lung function decline 1 year prior to anti‐fibrotic treatment (mean, 95% CI)	Rate of lung function decline 1 year following anti‐fibrotic treatment (mean, 95% CI)
FVC			
IPF (*n =* 396)	3.133 L (3.044–3.222)	158 mL (79–239)	38 mL (24–101)
PPF (*n =* 142)	2.651 L (2.505–2.797)	412 mL (308–517)	18 mL (9–124)
FVC (% predicted)			
IPF (*n =* 396)	83.27 (81.34–85.01)	0.98 (1.26–3.21)	0.81 (0.56–2.55)
PPF (*n =* 142)	71.30 (68.13–74.47)	8.98 (6.08–11.98)	1.03 (0.84–2.68)
DLCO (% predicted)			
IPF (*n =* 374)	49.00 (47.55–50.45)	9.42 (7.44–11.40)	2.55 (0.98–4.12)
PPF (*n =* 133)	40.77 (38.39–43.15)	8.71 (13.90–3.53)	0.94 (0.75–3.63)

Abbreviations: CI, confidence interval; DLCO, diffusing capacity of the lung for carbon monoxide; FVC, forced vital capacity; IPF, idiopathic pulmonary fibrosis; PPF, progressive pulmonary fibrosis other than IPF.

**FIGURE 1:**
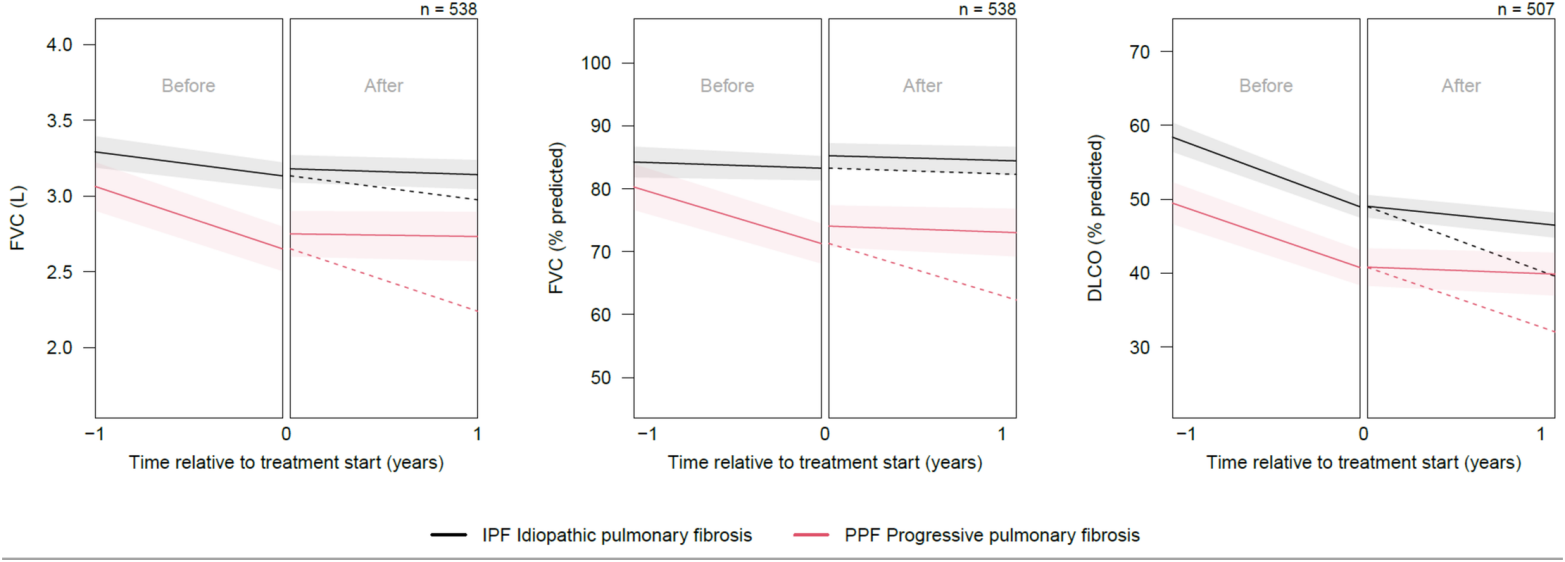
Estimated average lung function trajectories, with corresponding 95% confidence intervals, in idiopathic and progressive pulmonary fibrosis before and after anti‐fibrotic treatment initiation. DLCO, diffusing capacity of the lung for carbon monoxide; FVC, forced vital capacity; IPF, idiopathic pulmonary fibrosis; PPF, progressive pulmonary fibrosis. Linear mixed‐effects model analysis of in‐hospital forced vital capacity (FVC) in both litres and percent predicted, and diffusion capacity of the lungs for carbon (DLCO) in percentage predicted from 1 year before and after treatment initiation. Black represents the patients diagnosed with idiopathic pulmonary fibrosis, and red demonstrates the patients with progressive pulmonary fibrosis. Annual changes in FVC, FVC percentage predicted, and DLCO percentage predicted are displayed before and during anti‐fibrotic treatment in the left, middle, and right panels, respectively. The dotted lines represent the anticipated disease course, without initiation of anti‐fibrotic treatment.

In PPF, the mean annualised decline in FVC before initiation of anti‐fibrotic treatment was 412 mL (95% CI: 308–517 mL; see Figure 1 and Table 2). After initiation of anti‐fibrotic treatment, the mean annualised decline was 18 mL (95% CI: 9–124 mL). In IPF, the mean annualised decline in FVC before initiation of anti‐fibrotic treatment was 158 mL (95% CI: 78–239 mL). In the year after starting anti‐fibrotic treatment, the mean decline was 38 mL (95% CI: 24–101 mL).

Both in PPF (*p* = 0.01) and IPF (*p* = 0.01) decline in FVC significantly slowed down after the start of anti‐fibrotic treatment. In PPF, the difference in lung function decline in the year before and after the start of anti‐fibrotic treatment was 400 mL, whereas it was 124 mL in IPF (*p* ≤ 0.001).

In the PPF cohort, the mean annualised DLCO decline before treatment was 8.71% predicted (95% CI: 3.53%–13.90% predicted). In the first year after the initiation of treatment, patients had a mean decline of 0.94% predicted (95% CI: 0.75%–3.63% predicted).

Patients with IPF had a mean annualised DLCO decline of 9.42% predicted (95% CI: 7.44%–11.40% predicted) before starting anti‐fibrotic treatment. After starting treatment, the annualised average decline was 2.55% predicted (95% CI: 0.98%–4.12% predicted).

There was a statistically significant difference between the decline in DLCO before and the decline after initiation of treatment in patients with PPF and IPF (*p <* 0.001), with no difference between the declines observed in PPF and IPF (*p =* 0.567).

### 3.3 Disease Progression Within 1 Year After Anti‐Fibrotic Treatment Initiation

In PPF, 21.1% of patients had an absolute FVC decline of ≥ 5% and 7.0% of patients had an absolute decline of ≥ 10% in FVC in the first year after anti‐fibrotic treatment. In IPF, 22.5% had an FVC decline of ≥ 5%, and 7.8% had a decline of ≥ 10% in FVC in the first year after initiation of anti‐fibrotic treatment. 5.6% of patients with PPF and 7.8% of patients with IPF had a decline of ≥ 10% in DLCO (Table 3).

**TABLE 3:** Proportion of patients with idiopathic pulmonary fibrosis and progressive pulmonary fibrosis—in the first year after initiation of anti‐fibrotic therapy.

	IPF (*n =* 396)	PPF (*n =* 142)
Absolute decline of ≥ 5% in FVC, % (*n*)	22.5 (89)	21.1 (30)
Absolute decline of ≥ 10% in FVC, % (*n*)	7.8 (31)	7.0 (10)
Absolute decline of ≥ 10% in DLCO, % (*n*)	7.8 (31)	5.6 (8)
Absolute decline of ≥ 5% in FVC and ≥ 10% DLCO, % (*n*)	2.3 (9)	2.8 (4)
Absolute decline of ≥ 10% in FVC and ≥ 10% DLCO, % (*n*)	0.8 (3)	1.4 (2)

Abbreviations: DLCO, diffusing capacity of the lung for carbon monoxide; FVC, forced vital capacity; IPF, idiopathic pulmonary fibrosis; PPF, progressive pulmonary fibrosis.

Four patients died within the first year after anti‐fibrotic treatment initiation (three patients with IPF, and one patient with PPF) and three patients underwent lung transplantation (two patients with IPF, and one patient with PPF).

## 4 Discussion

In this nation‐wide registry study, there was a significant reduction in lung function decline in both PPF and IPF in the year following the start of anti‐fibrotic treatment. Nevertheless, a significant proportion of patients still met the physiological criteria of disease progression.

In this large real‐world cohort of patients with PPF and IPF, we observed a change in decline after the initiation of anti‐fibrotic treatment, in line with other real‐world cohort studies. Raman and colleagues [12] have reported a mean FVC decline of 89 mL in the year after the start of nintedanib, compared with a decline of 240 mL in the year prior to treatment initiation in the groups of patients with different forms of PPF. Similar real‐world data are available for IPF [14, 15, 16]. We found that the lung function courses of patients with IPF and PPF after the initiation of anti‐fibrotic treatment were similar. However, our findings revealed a notably lower FVC at the onset of anti‐fibrotic treatment in patients with PPF compared with IPF, and a significant difference in slope before treatment, with PPF having a greater decline in FVC. One of the reasons for this is that the included non‐IPF patients were initially treated with immunosuppression prior to the start of anti‐fibrotics. They had to deteriorate in order for nintedanib to be initiated. In IPF, anti‐fibrotics are started in an earlier phase of the disease. Thus, the non‐IPF group is a worse group compared to the IPF cohort. Furthermore, anti‐fibrotic treatment was not approved for use in PPF before July 2020 in the Netherlands, and only a small number of patients were treated earlier in a clinical trial setting. Hence, patients with PPF may have been progressive for a longer period of time before receiving anti‐fibrotic treatment than those with IPF. Although awareness of differences in disease behaviour in PPF is increasing, there may still have been a lack of awareness and delays in detecting disease progression. The coming years will determine if this access effect weans out and if patients with PPF will be treated earlier.

As lower baseline FVC has been shown to be associated with an increased risk of subsequent mortality in different forms of pulmonary fibrosis [17, 18], early detection of disease progression and early treatment initiation are most important. Care access is, however, pressured, and new strategies are needed to monitor patients. A novel way of identifying disease progression at an early stage is through online home monitoring, which would allow for frequent monitoring of FVC without the burden of additional hospital visits [19]. Furthermore, serum and HRCT biomarkers may also help predict which patients are at risk for progression [20, 21, 22, 23]. Unfortunately, none of these techniques have yet made it to broad clinical implementation.

To our knowledge, this is the first real‐world study that evaluated the proportion of patients with PPF who were progressive during anti‐fibrotic treatment. Studies in IPF have demonstrated that around 10% of patients have disease progression in the first year after treatment [24, 25]. However, in real‐world data from registry studies, these percentages fluctuate, with 38% the highest reported [26]. The differences between these study results may be partially explained by the different criteria that were used to classify disease progression. In this study, we did not have access to radiology or data on the worsening of respiratory symptoms for all patients. Therefore, we only used the physiological criteria from the recent ATS/ERS/JRS/ALAT guidelines to define progression. This implies that the real percentage of patients with disease progression in our cohort was likely higher than 24%. Nevertheless, we know from previous studies that a decline in FVC is the criterion most often used to define disease progression, suggesting that we identified the majority of patients with disease progression in the year after initiation of anti‐fibrotic treatment [27]. Our study shows that there is a minority of patients with PPF who showed progression within the first year after starting anti‐fibrotic treatment. This is the patient group that should be identified to offer participation in clinical trials for novel compounds on top of anti‐fibrotic medication. As many of the current studies use the relative decline in FVC in the past 2 years as part of their inclusion criteria, the group of patients who are eligible for studies may be even larger [28, 29].

An area for ongoing research is the identification of factors that predict response to immunosuppressive and anti‐fibrotic treatment [30]. A clinical challenge in many patients with PPF is deciding whether and how to proceed with immunosuppressive treatment once anti‐fibrotic treatment is started. In our PPF cohort, two‐thirds used concurrent immunosuppressive medication, with a small number of medication switches within the first year following the initiation of anti‐fibrotic treatment, but with almost half the patients discontinuing their immunosuppressive treatment. Our cohort is too small and heterogeneous to perform subgroup analysis of the lung function in patients that continued immunosuppression next to anti‐fibrotic treatment and those that discontinued.

This study has some limitations. First, this is an observational study wherein patients with PPF serve as their own controls. Therefore, the results should be interpreted with caution. However, our results are in line with other published studies and contribute to the accumulating evidence on the effect of anti‐fibrotics in PPF [12, 13]. Furthermore, the data on lung function and medication in the NVALT registry were collected retrospectively for the period before the initiation of anti‐fibrotic medication. Moreover, although this was a large real‐world PPF cohort, our study is still lacking power to provide a subgroup analysis to determine FVC decline in individual diseases for the different medication regimens. Future studies should emphasise providing insights into the impact of combining different immunosuppressive regimens with anti‐fibrotic treatment on the course of lung function in various diseases. Additionally, our inclusion period was between 2022 and 2023, which meant that patients had to be alive at least in 2022, which has introduced a survival bias. Lastly, patients were included based on the physiological criteria as stated in the INBUILD trial [6]. Although a form of selection bias may have been introduced, we do believe that its impact is limited as a minority of subjects in the INBUILD trial were included solely based on respiratory symptoms and radiological criterion [31].

In conclusion, this multicentre real‐world registry study shows that in the majority of patients with PPF and IPF, FVC decline significantly decreased in the year after initiation of anti‐fibrotic treatment. However, there is a subgroup of patients who do not benefit enough from current treatment strategies. Future studies should aim to gain deeper insights into this specific patient group and identify prognostic factors to determine at an early stage which patients might benefit from anti‐fibrotic treatment or specific combinations of anti‐fibrotic and immunosuppressive treatment.

## Author Contributions

**Mark G. J. P. Platenburg:** conceptualization (lead), data curation (equal), formal analysis (equal), investigation (equal), methodology (lead), resources (equal), writing – original draft (equal), writing – review and editing (equal). **Gizal Nakshbandi:** conceptualization (equal), data curation (equal), formal analysis (equal), investigation (equal), methodology (equal), resources (equal), validation (equal), visualization (equal), writing – original draft (equal), writing – review and editing (equal). **Catharina C. Moor:** conceptualization (equal), project administration (equal), supervision (equal), writing – review and editing (equal). **Aernoud A. van Batenburg:** project administration (equal), resources (equal), software (equal), writing – review and editing (equal). **Rémy L. M. Mostard:** supervision (equal), writing – review and editing (equal). **Mareye Voortman:** supervision (equal), writing – review and editing (equal). **Linda A.A. Moonen:** supervision (equal), writing – review and editing (equal). **Nicolle Hekelaar:** supervision (equal), writing – review and editing (equal). **Maria J. Overbeek:** supervision (equal), writing – review and editing (equal). **Brigitte A.H.A. Bogaarts:** supervision (equal), writing – review and editing (equal). **Henk Kramer:** supervision (equal), writing – review and editing (equal). **Emiel. R. Marges:** supervision (equal), writing – review and editing (equal). **Bart B. Boerrigter:** supervision (equal), writing – review and editing (equal). **Paul Bresser:** supervision (equal), writing – review and editing (equal). **Eveline L. Schakenraad:** supervision (equal), writing – review and editing (equal). **Jan van der Maten:** supervision (equal), writing – review and editing (equal). **Niels C.A. van der Sloot:** supervision (equal), writing – review and editing (equal). **Stefan Walen:** supervision (equal), writing – review and editing (equal). **Pedro Miranda Afonso:** investigation (equal), writing – review and editing (equal). **Marlies S. Wijsenbeek:** funding acquisition (equal), supervision (equal), writing – review and editing (equal). **Jan C. Grutters:** project administration (equal), supervision (equal), writing – review and editing (equal).

## Ethics Statement

The study was performed in accordance with the ethical principles of the Declaration of Helsinki and approved by the Medical research Ethics committees United Nieuwegein, the Netherlands, METC number: W20.228. All patients signed an informed consent for the use of their data for research purposes.

## Conflicts of Interest

This registry was funded by Boehringer Ingelheim.

## Data Availability

Data that support these findings are available from the corresponding author upon reasonable request.
